# The Extent of Ventilator-Induced Lung Injury in Mice Partly Depends on Duration of Mechanical Ventilation

**DOI:** 10.1155/2013/435236

**Published:** 2013-04-17

**Authors:** Maria A. Hegeman, Sabrine N. T. Hemmes, Maria T. Kuipers, Lieuwe D. J. Bos, Geartsje Jongsma, Joris J. T. H. Roelofs, Koenraad F. van der Sluijs, Nicole P. Juffermans, Margreeth B. Vroom, Marcus J. Schultz

**Affiliations:** ^1^Laboratory of Experimental Intensive Care and Anesthesiology (LEICA), Academic Medical Center, Meibergdreef 9, 1105 AZ Amsterdam, The Netherlands; ^2^Department of Pathology, Academic Medical Center, Meibergdreef 9, 1105 AZ Amsterdam, The Netherlands; ^3^Department of Intensive Care, Academic Medical Center, Meibergdreef 9, 1105 AZ Amsterdam, The Netherlands

## Abstract

*Background*. Mechanical ventilation (MV) has the potential to initiate ventilator-induced lung injury (VILI). The pathogenesis of VILI has been primarily studied in animal models using more or less injurious ventilator settings. However, we speculate that duration of MV also influences severity and character of VILI. *Methods*. Sixty-four healthy C57Bl/6 mice were mechanically ventilated for 5 or 12 hours, using lower tidal volumes with positive end-expiratory pressure (PEEP) or higher tidal volumes without PEEP. Fifteen nonventilated mice served as controls. *Results*. All animals remained hemodynamically stable and survived MV protocols. In both MV groups, PaO_2_ to FiO_2_ ratios were lower and alveolar cell counts were higher after 12 hours of MV compared to 5 hours. Alveolar-capillary permeability was increased after 12 hours compared to 5 hours, although differences did not reach statistical significance. Lung levels of inflammatory mediators did not further increase over time. Only in mice ventilated with increased strain, lung compliance declined and wet to dry ratio increased after 12 hours of MV compared to 5 hours. *Conclusions*. Deleterious effects of MV are partly dependent on its duration. Even lower tidal volumes with PEEP may initiate aspects of VILI after 12 hours of MV.

## 1. Introduction

Increased strain due to mechanical ventilation (MV) has the potential to aggravate existing lung injury [1]. Indeed, one meta-analysis shows intensive care unit (ICU) patients with acute respiratory distress syndrome (ARDS) to benefit from MV with lower tidal volume V_T_ [2]. MV with too high V_T_ even has the potential to induce lung injury [3]. This is confirmed in a more recent meta-analysis that shows patients without ARDS at onset of MV to benefit from MV with lower V_T_ as well [4]. Importantly, this meta-analysis also showed beneficial effects of lower V_T_ in patients receiving MV during general anesthesia for surgery [4].

The potential of MV to aggravate or initiate lung injury was originally proposed in animal models and focused merely on size of V_T_. Indeed, the so-called ventilator-induced lung injury (VILI) was demonstrated in models of MV in animals with injured lungs [5]. These models revealed that use of high V_T_ worsened the proinflammatory response, disturbed alveolar fibrin turnover, and increased alveolar-capillary permeability resulting in accumulation of protein-rich edema and finally loss of pulmonary function. VILI was also observed in ventilated animals with noninjured lungs [6–10], confirming clinical studies, which suggest that conventional MV has the capability to initiate lung injury by itself. Most interestingly, even MV with lower V_T_ is recently found to induce VILI in healthy animals [11–13].

Animal models with variable durations of MV are important for preclinical testing of ventilator settings, as duration of surgical procedures may vary significantly. Moreover, a vast number of patients may need additional postoperative MV, especially after major surgery. Although the evolution of VILI has been studied even beyond 24 hours in large animal models [14, 15], studies testing the effect of duration of MV on development of VILI are limited in smaller animals like mice. One important advantage of mice above larger animals is the possible application of transgenic or knockout models. Therefore, the aim of the present study was to compare the effects after 12 hours of MV with those after 5 hours in an established model of VILI in healthy mice, that is, without preexisting lung injury. Different V_T_ and positive end-expiratory pressure (PEEP) levels were used to create two opposing ventilation strategies, a strategy with lower V_T_ and PEEP (LV_T_/PEEP) or a strategy with higher V_T_ and zero PEEP (HV_T_/ZEEP). We hypothesized that the deleterious effects of MV are not only dependent on its strategy but also on its duration.

## 2. Methods

### 2.1. Approval

The animal care and use committee of the Academic Medical Center, Amsterdam, the Netherlands, approved all experiments. Animal handling was in accordance with institutional standards for care and use of laboratory animals.

### 2.2. Animals

Seventy-nine male C57Bl/6 mice (26–30 grams) were randomly assigned to different experimental groups. Sixty-four mice were randomized to MV and fifteen mice were randomized to nonventilated controls (NVC). All mice were without preexisting lung injury at time of randomization.

### 2.3. Animal Handling

Mice received an intraperitoneal bolus of 1 mL 0.9% saline. After 1 hour, mice were randomized to MV or NVC. Mice that were randomized to MV received an induction of anesthesia via intraperitoneal injection of a mix containing 126 mg/kg ketamine (Eurovet Animal Health B.V., Bladel, the Netherlands), 0.1 mg/kg dexmedetomidine (Pfizer Animal Health B.V., Capelle aan den IJssel, the Netherlands) and 0.5 mg/kg atropine (Pharmachemie, Haarlem, the Netherlands). Maintenance anesthesia was administered via an intraperitoneal cathether every hour and consisted of 36 mg/kg ketamine, 0.02 mg/kg dexmedetomidine and 0.075 mg/kg atropine. Sodium bicarbonate was administered via an intraperitoneal cathether every 30 minutes to maintain bicarbonate levels within the physiological range (22–26 mM). No muscle relaxants were used. Body temperature was kept between 36.5 and 37.5°C. 

### 2.4. Mechanical Ventilation

After insertion of a tracheotomy tube (1.3 mm outer diameter and 0.8 mm inner diameter), mice were connected to a Babylog 8000 plus ventilator (Draeger Medical, Lubeck, Germany) and mechanically ventilated for 5 or 12 hours using a pressure-controlled, volume-targeted approach, at a fractional inspired oxygen concentration (FiO_2_) of 0.5 and an inspiration-to-expiration ratio of 1 : 3. A pneumotachograph was used for monitoring and continuous regulation of V_T_ (capillary tube, PTM T16375; HSE-Harvard Apparatus, March-Hugstetten, Germany). V_T_ was recorded using respiration software (HSE-BDAS basic data acquisition, HSE-Harvard Apparatus); delivered pressure was regularly adapted to deliver target V_T_. 

### 2.5. Study Groups

Mice that were randomized to MV were mechanically ventilated with lower V_T_ (~7 mL/kg) and PEEP of 3 cmH_2_O (LV_T_/PEEP) or with higher V_T_ (~15 mL/kg) and PEEP of 0 cmH_2_O (HV_T_/ZEEP). Respiratory rate was set at 160 or 52 breaths per minute, respectively, aiming at normal pH (7.35–7.45). A recruitment maneuver was performed every 30 minutes during LV_T_/PEEP and every 60 minutes during HV_T_/ZEEP by applying an inspiratory hold for 5 seconds, with increased inspiratory pressures when necessary, aiming at normal PaCO_2_ (35–45 mmHg). The last recruitment maneuver was performed 30 or 60 minutes before blood sampling (LV_T_/PEEP and HV_T_/ZEEP, resp.), which was similar in mice ventilated for 5 or 12 hours.

### 2.6. Monitoring

Systolic blood pressure and heart rate were noninvasively monitored using a tail-cuff system for mice (ADInstruments, Spenbach, Germany). Peripheral oxygen saturation (SpO_2_) was noninvasively measured using a pulse oximeter applied to the mouse hind paw (Siemens Medical Systems, Danvers, MA, USA). After 5 or 12 hours of MV, arterial blood was taken from the carotid artery for blood gas analysis (RAPIDPoint 405; Siemens Healthcare Diagnostics, Tarrytown, NY, USA).

Compliance of the respiratory system was calculated using *C*_stat_ = V_ T _/(*P*_plat_ − PEEP), in which *C*_stat_ is the static compliance (mL/cmH_2_O), and *P*_plat_ is the plateau pressure (cmH_2_O). V_T_ was determined using the pneumotachograph. *P*_plat_ and PEEP were displayed on the mechanical ventilator. The respiration software revealed a decelerating flow curve during both inspiration and expiration, and a square-wave pressure curve (hourly monitored).

### 2.7. Lung Tissue

Lung tissue was harvested and processed as previously described [13, 16]. From a first series of mice (*n* = 6–8 per group), the right lung was used to obtain bronchoalveolar lavage fluid (BALF) and the left lung was used for wet to dry ratios. From a second series of mice (*n* = 6–8 per group), the right lung was snap frozen to obtain lung homogenates and the left lung used for histopathology.

### 2.8. Assays

Interleukin (IL)-1*β*, IL-6, keratinocyte-derived chemokine (KC), and macrophage inflammatory protein- (MIP)-2 levels were measured in total lung homogenates and receptor for advanced glycation endproducts (RAGE) levels were measured in BALF by ELISA (R&D systems, Minneapolis, MN, USA). Total protein levels were determined in BALF using a Bradford Protein Assay Kit according to manufacturer's instructions with bovine serum albumin as standard (OZ Biosciences, Marseille, France). Immunoglobulin (Ig)M levels were measured in BALF by ELISA as previously described [17].

### 2.9. Statistical Analysis

Data are presented as median (IQR) or scatter plot (median), as appropriate. Since group characteristics did not follow a normal distribution, differences between groups were analyzed by Kruskal-Wallis tests with post hoc Mann-Whitney tests and Bonferroni correction. We first compared 12 hours of MV with 5 hours or NVC (*P* value for significance was set at 0.0125); next we compared LV_T_/PEEP with HV_T_/ZEEP ventilation at 12 hours (*P* value for significance was set at 0.01). Seven mice were excluded from analysis because of various reasons (i.e., blood in BALF (*n* = 4), unstable blood pressure (*n* = 1), and unreliable cell count measurement [*n* = 2]).

## 3. Results

### 3.1. Hemodynamic and Respiratory Parameters

**Figure 1:**
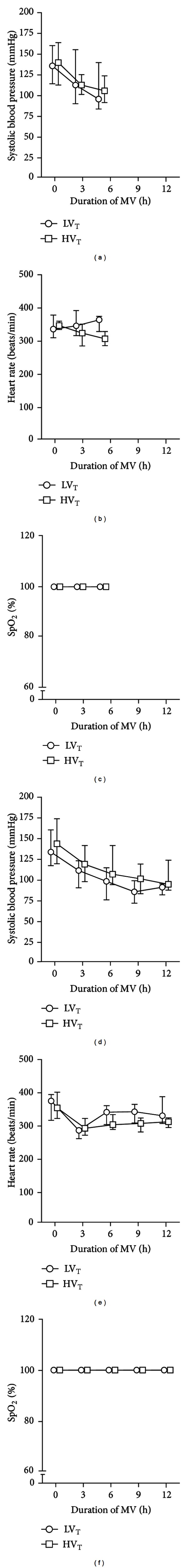
Hemodynamic parameters. Systolic blood pressures, heart rates, and peripheral oxygen saturation (SpO_2_) remained stable throughout 5 hours ((a)–(c)) and 12 hours ((d)–(f)) of mechanical ventilation (MV). Data are presented as median (IQR) of 12–15 ((a)-(b), (d)-(e)), or 3–6 ((c), (f)) mice per group (circle = LV_T_; square = HV_T_). LV_T_, HV_T_ = LV_T_/PEEP or HV_T_/ZEEP ventilator settings.

**Figure 2:**
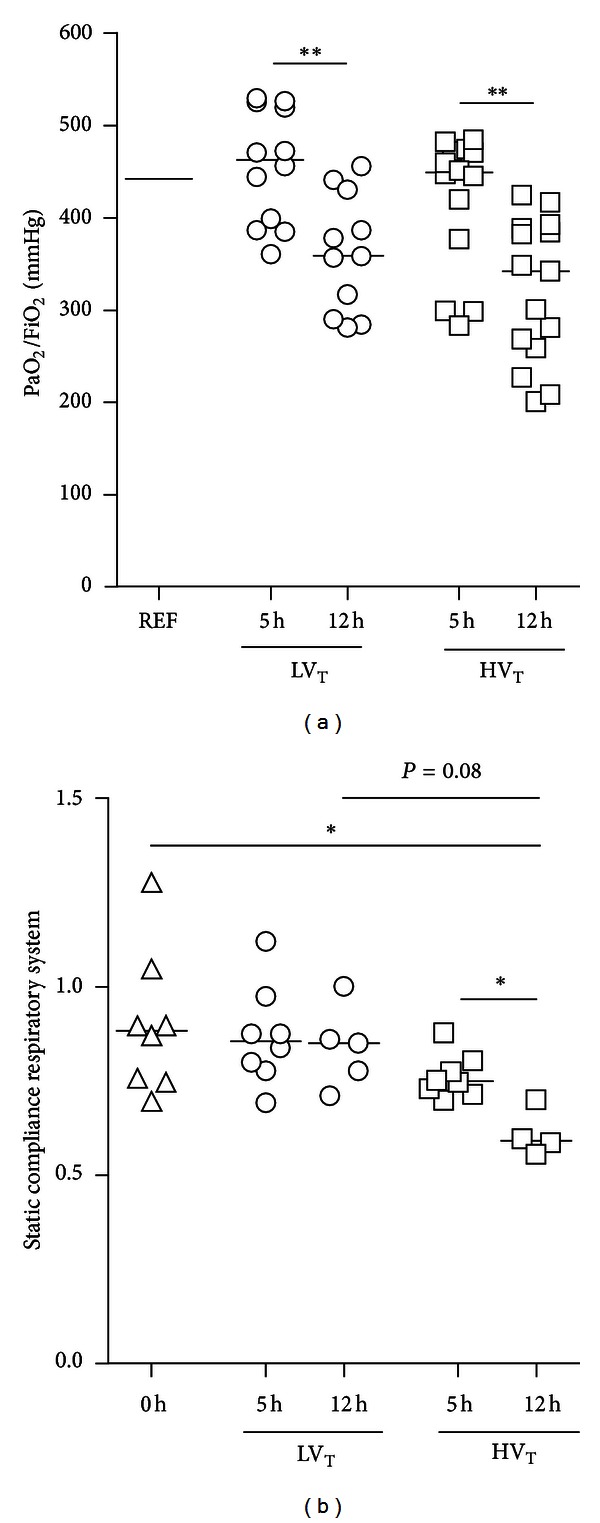
Respiratory parameters. Lung function is represented by the PaO_2_ to FiO_2_ ratio (PaO_2_/FiO_2_) (a) and static compliance of the respiratory system (b). The reference (REF) represents the median PaO_2_/FiO_2_ from 7 mice ventilated with LV_T_/PEEP for 30 minutes, that is, 442.4 mmHg (396.2 to 501.0) (a) or the combined static compliance of LV_T_/PEEP and HV_T_/ZEEP-ventilated mice at *t* = 0 h (b). The static compliance did not differ between the two groups at *t* = 0 h. Data are presented as scatter plot (median) of 11–15 (a) or 4–8 (b) mice per group (triangle = REF; circle = LV_T_; square = HV_T_). *Illustrates primary statistical analysis (*P < 0.05, **P < 0.01). NVC = nonventilated controls; LV_T_, HV_T_ = LV_T_/PEEP or HV_T_/ZEEP ventilator settings; 5 h, 12 h = 5 or 12 hours of ventilation.

All mice were ventilated in a pressure-controlled, volume-targeted approach. In LV_T_/PEEP ventilated mice, V_T_ was maintained at 7.0 mL/kg by delivering a *P*_plat_ of 11.0 cmH_2_O throughout 12 hours of MV (Table 1(A)). In HV_T_/ZEEP ventilated mice, V_T_ was maintained at 15.0 mL/kg by delivering a *P*_plat_ of 20.0 cmH_2_O at 5 hours of MV increasing to 25.5 cmH_2_O at 12 hours. All animals survived the experimental procedures throughout 5 or 12 hours of MV. Systolic blood pressures and heart rates remained stable and SpO_2_ levels remained ≥90% during 5 or 12 hours of MV, independent of ventilation strategy (Figure 1). PaCO_2_, pH, base excess, and HCO_3_^−^ levels remained within normal to near-normal range in all series of experiments (Table 1(B)). In both MV groups, PaO_2_ to FiO_2_ ratios were lower after 12 hours of MV compared to 5 hours (Figure 2(a)). Lung compliances were also lower after 12 hours of MV compared to 5 hours in mice ventilated with HV_T_/ZEEP, but not in mice ventilated with LV_T_/PEEP (Figure 2(b)).

### 3.2. Edema Formation and Alveolar-Capillary Permeability

**Figure 3:**
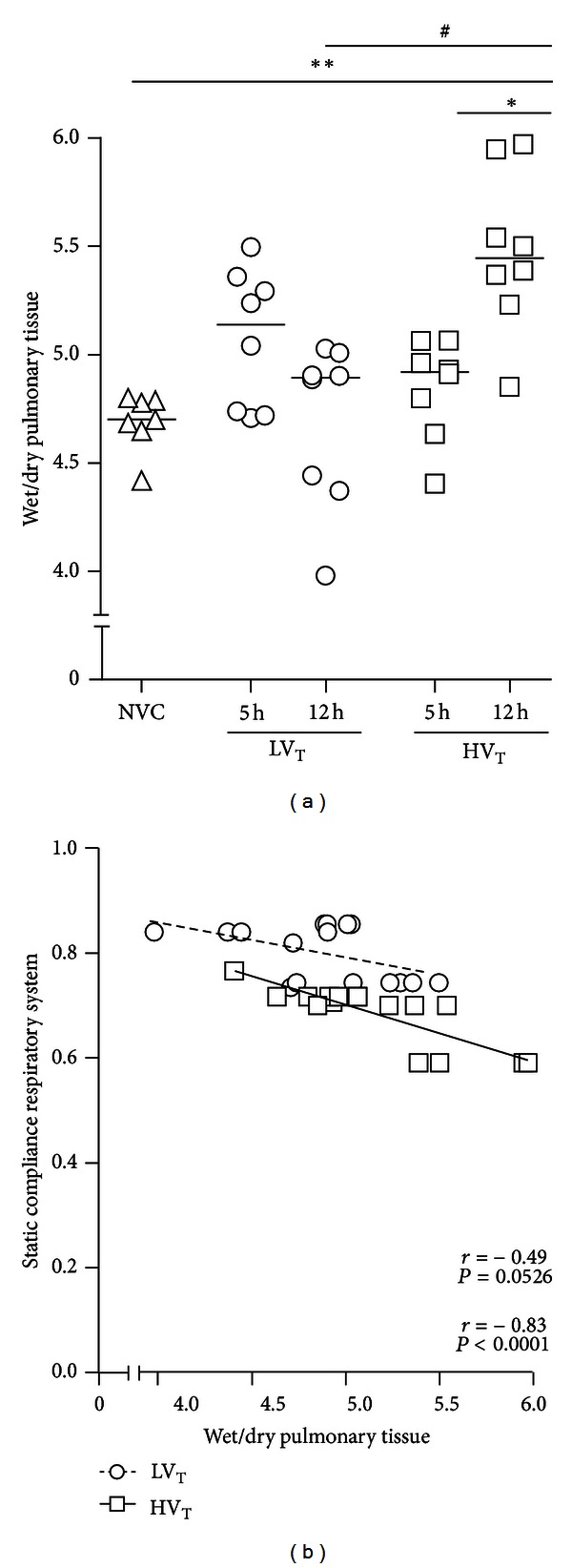
Edema formation. (a): Edema formation is represented by wet to dry ratios of lung tissue (Wet/Dry). Data are presented as scatter plot (median) of 7-8 mice per group (triangle = NVC; circle = LV_T_; square = HV_T_). *Illustrates primary statistical analysis (*P < 0.05, **P < 0.01); ^#^illustrates secondary statistical analysis (^#^ *P* < 0.05). (b) In LV_T_/PEEP and HV_T_/ZEEP-ventilated mice, correlation analyses were performed between static compliance of respiratory system and wet/dry of pulmonary tissue. Linear correlations, Pearson correlation coefficients (*r*), and *P* values are depicted. NVC = nonventilated controls; LV_T_, HV_T_ = LV_T_/PEEP or HV_T_/ZEEP ventilator settings; 5 h, 12 h = 5 or 12 hours of ventilation.

**Figure 4:**
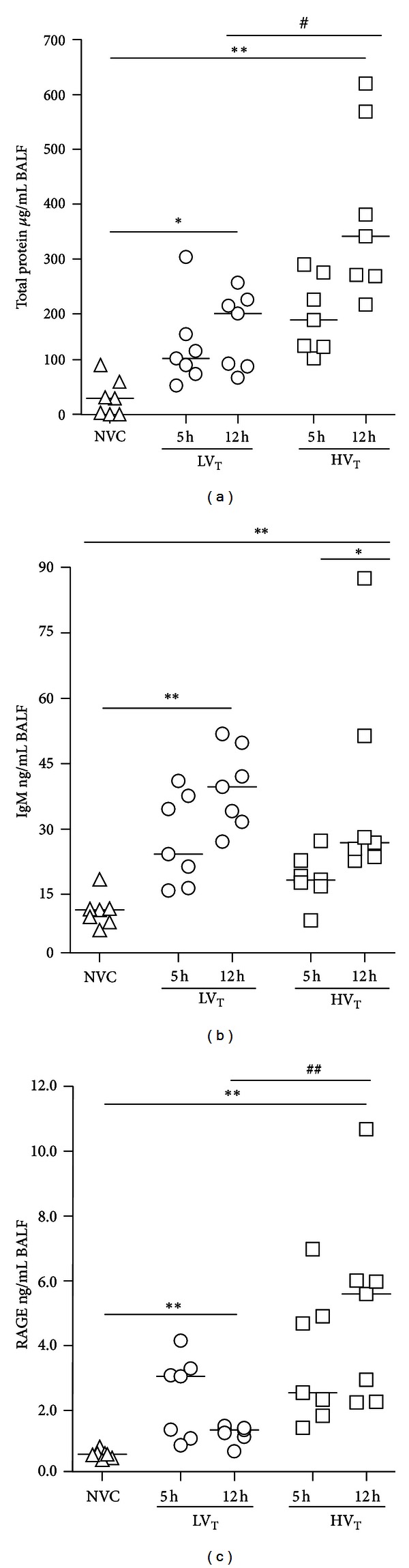
Alveolar-capillary permeability. ((a)-(b)) Alveolar-capillary permeability is represented by total protein and immunoglobulin (Ig)M levels in bronchoalveolar lavage fluid (BALF). (c) Alveolar epithelial type 1 cell injury is represented by receptor for advanced glycation end-products (RAGE) levels in BALF [18]. Data are presented as scatter plot (median) of 7-8 mice per group (triangle = NVC; circle = LV_T_; square = HV_T_). *Illustrates primary statistical analysis (*P < 0.05, **P < 0.01); ^#^Illustrates secondary statistical analysis (^#^ *P* < 0.05, ^##^ *P* < 0.01). NVC = nonventilated controls; LV_T_, HV_T_ = LV_T_/PEEP or HV_T_/ZEEP ventilator settings; 5 h, 12 h = 5 or 12 hours of ventilation.

Lung wet to dry ratios were higher after 12 hours of MV compared to 5 hours in mice ventilated with HV_T_/ZEEP, but not in mice ventilated with LV_T_/PEEP (Figure 3(a)). Lung wet to dry ratios showed a negative correlation with lung compliances, especially in HV_T_/ZEEP-ventilated mice (Figure 3(b)). BALF total protein, IgM, and RAGE levels tended to be higher after 12 hours of MV compared to 5 hours in both ventilation groups, although only with statistical significance for IgM in mice ventilated with HV_T_/ZEEP (Figures 4(a)–4(c)).

### 3.3. Cell Infiltration

**Figure 5:**
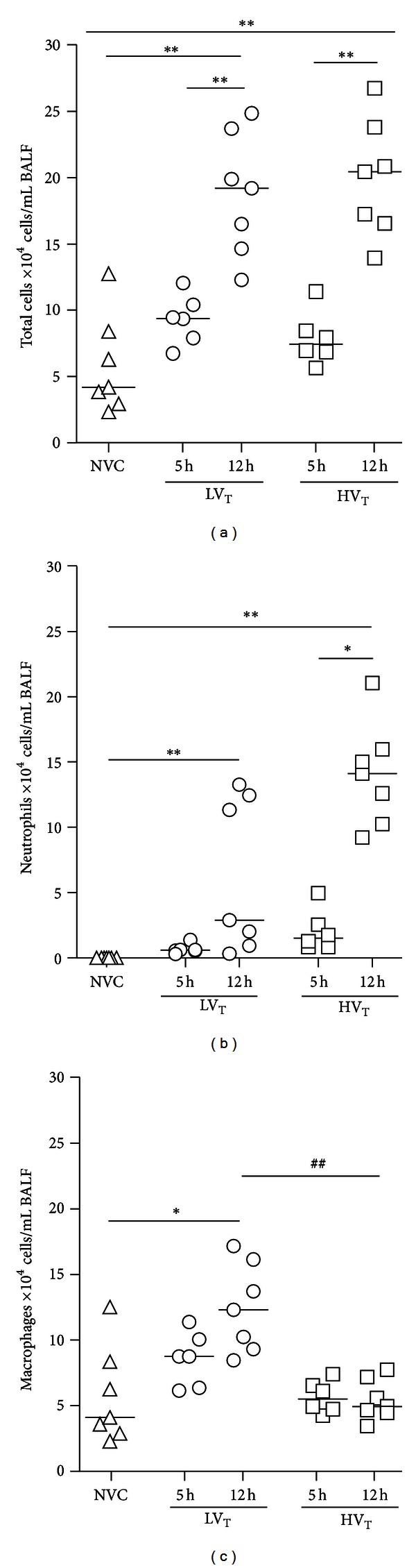
Cell infiltration. (a) Total cell counts were measured in bronchoalveolar lavage fluid (BALF). ((b)-(c)) Differential cell counts were performed on BALF cytospin preparations to determine neutrophil and macrophage infiltration. Data are presented as scatter plot (median) of 6–8 mice per group (triangle = NVC; circle = LV_T_; square = HV_T_). *Illustrates primary statistical analysis (*P < 0.05, **P < 0.01); ^#^illustrates secondary statistical analysis (^##^ *P* < 0.01). NVC = nonventilated controls; LV_T_, HV_T_ = LV_T_/PEEP or HV_T_/ZEEP ventilator settings; 5 h, 12 h = 5 or 12 hours of ventilation.

BALF cell contents were elevated after 12 hours of MV compared to 5 hours, independent of ventilation strategy (Figure 5(a)). BALF neutrophil counts were higher after 12 hours of MV compared to 5 hours in both ventilation groups, although differences did not reach statistical significance when comparing 12 with 5 hours of MV in mice ventilated with LV_T_/PEEP (Figure 5(b)). BALF macrophage counts were elevated after 12 hours of MV compared to 5 hours in mice ventilated with LV_T_/PEEP, but not in mice ventilated with HV_T_/ZEEP (Figure 5(c)).

### 3.4. Inflammatory Mediators

**Figure 6:**
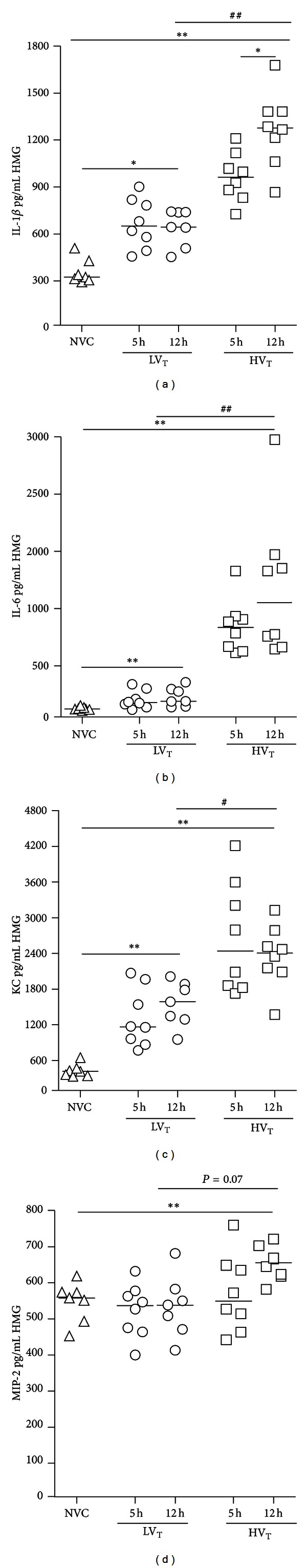
Inflammatory mediators. ((a)-(b)) Protein levels of the proinflammatory cytokines interleukin (IL)-1*β* and IL-6 were determined in supernatant of total lung homogenates (HMG). ((c)–(d)) In addition, protein levels of the chemotactic cytokines keratinocyte-derived chemokine (KC) and macrophage inflammatory protein (MIP)-2 were determined. Data are presented as scatter plot (median) of 7-8 mice per group (triangle = NVC; circle = LV_T_; square = HV_T_). *Illustrates primary statistical analysis (*P < 0.05, **P < 0.01); ^#^illustrates secondary statistical analysis (^#^ *P* < 0.05, ^##^ *P* < 0.01). NVC = nonventilated controls; LV_T_, HV_T_ = LV_T_/PEEP or HV_T_/ZEEP ventilator settings; 5 h, 12 h = 5 or 12 hours of ventilation.

Lung IL-1*β*, IL-6, KC, and MIP-2 levels increased after 12 hours of MV compared to NVC in both ventilation groups, except for MIP-2 levels in mice ventilated with LV_T_/PEEP (Figures 6(a)–6(d)). In addition, lung IL-1*β* and MIP-2 levels were higher after 12 hours of MV compared to 5 hours in mice ventilated with HV_T_/ZEEP, although differences in MIP-2 levels did not reach statistical significance (Figures 6(a) and 6(d)).

### 3.5. Lung Histopathology

**Figure 7:**
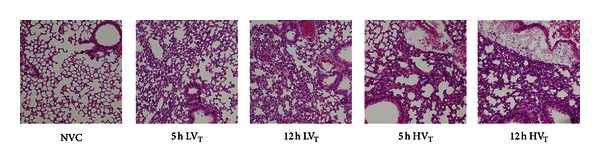
Histopathology. Lung sections were stained with hematoxylin eosin (H&E) to analyze lung histopathology (magnification 100x). Total histopathology score was determined by the sum of the score for 4 pathologic parameters: (a) edema, (b) hemorrhage, (c) interstitial cell infiltration, and (d) hyaline membranes. NVC = nonventilated controls; LV_T_, HV_T_ = LV_T_/PEEP or HV_T_/ZEEP ventilator settings; 5 h, 12 h = 5 or 12 hours of ventilation.

Histopathological changes due to MV were minor and were recognizable as edema formation and interstitial infiltration of inflammatory cells (Figure 7). Differences in total histopathology score were only observed between 12 hours of HV_T_/ZEEP ventilation and NVC (Table 2).

### 3.6. Differences between MV Strategies

Differences between the two ventilation groups after 12 hours of MV confirm previous findings, with more lung injury with HV_T_/ZEEP as compared to LV_T_/PEEP ventilation. These differences include lung wet to dry ratios (Figure 3(a)), BALF total protein levels (Figure 4(a)), BALF RAGE levels (Figure 4(c)), lung IL-1*β* levels (Figure 6(a)), lung IL-6 levels (Figure 6(b)), lung KC levels (Figure 6(c)), and total histopathology score (Table 2). In contrast, BALF macrophage numbers were higher after 12 hours of LV_T_/PEEP ventilation compared to 12 hours of HV_T_/ZEEP ventilation (Figure 5(c)).

## 4. Discussion

The present study shows that the appearance of VILI depends not only on the strategy but also on the duration of MV. Indeed, well-known characteristics of VILI evolved over time, with longer duration of MV having a greater effect in strategies with HV_T_/ZEEP than in strategies with LV_T_/PEEP. Moreover, lung injury is even caused by less injurious MV settings when extending the duration.

The results of the present study are, at least in part, in line with previous clinical and animal studies showing that MV has the potential to cause lung injury in healthy lungs. Indeed, two retrospective studies of patients without ARDS at onset of MV suggest that MV with high V_T_ is a risk factor for developing lung injury [19, 20]. A more recent randomized controlled trial provides additional evidence by showing that MV with lower V_T_ prevents lung injury in critically ill patients without ARDS at onset of MV [21]. Previous animal studies confirmed that mice with noninjured lungs can develop VILI when exposed to MV [6–10]. Thus, preexisting lung injury is not a prerequisite for the devastating effects of MV. The current finding that even less injurious MV settings can cause lung injury is in line with previous animal studies [11–13]. It should be noted that the majority of small animal investigations studied the effects of MV over relatively short durations. Our data in mice show that the phenotype of VILI changes with duration of MV. Alveolar-capillary barrier dysfunction and inflammation are early features of VILI. Decrease in PaO_2_ to FiO_2_ ratios is observed after a longer duration of MV, whereas neutrophil infiltration was most pronounced after 12 hours of MV. These findings suggest that development of VILI not only progresses but also evolves over time. Thus, small animal investigations using shorter-lasting MV may have underestimated the severity and time-dependent character of VILI. In large animal models, the evolution of VILI beyond 24 hours has been described before [14, 15].

There is convincing evidence that even MV during general anesthesia for surgery has the potential to initiate subtle pulmonary changes [22–26]. In addition, postoperative pulmonary complications add to the morbidity and mortality of surgical patients [27, 28] and clinical studies suggest that less injurious MV settings in the perioperative period may reduce postoperative respiratory morbidity [24, 29–31]. As smaller animals have different respiratory mechanisms than humans [32, 33] and are less resistant to VILI [34], it should be taken into account that the effect of MV in the experimental setting may not be completely comparable to the clinical setting. Considering the duration of MV used in animal models so far, one could argue that current animal models better reflect the clinical scenario of patients who require general anesthesia for surgery than those who require intensive care. In view of this notion, experimental studies using longer durations of MV may therefore mimic the clinical scenario of patients who need MV for longer-lasting surgical procedures, or patients who need postoperative MV for several hours.

Previous clinical studies clearly show that it makes a difference as far which ventilator settings are being used during the perioperative phase of major surgery [23, 35]. Although clinical trials about the effects of ventilation strategies in the postoperative setting are lacking, it has been suggested that the use lower V_T_ should be considered in all mechanically ventilated patients [3]. Present experimental data may contribute to our understanding of optimal ventilator strategies in patients who need postoperative MV for several hours. This study confirms that extent of VILI is dependent on the used V_T_. In addition, this study demonstrates that 5 hours of MV may not be as detrimental as 12 hours of MV. So, it may be important to consider that the aspects of VILI are not only critically influenced by V_T_, but also by duration of MV. Indeed, 12 hours of LV_T_/PEEP ventilation appeared to induce important aspects of VILI as well. Interestingly, increased macrophage numbers were observed after 12 hours of LV_T_/PEEP ventilation but not after 12 hours of HV_T_/ZEEP ventilation. The failure to recover BALF macrophages after 12 hours of HV_T_/ZEEP could suggest macrophage activation and adhesion to lung tissue which may account for orchestrating the increase in proinflammatory mediators and recruitment of neutrophils. Recent studies, however, revealed the importance of macrophages in the termination and resolution of inflammation [36]. Therefore, an alternative explanation is that the presence of more macrophages after 12 hours of LV_T_/PEEP ventilation could play a protective role in the development of VILI. It has been previously shown that macrophages are involved in tissue repair and as a result capable of restoring lung barrier integrity [36]. Supporting the latter explanation, a negative correlation was found between BALF macrophage numbers and wet to dry ratios in LV_T_/PEEP-ventilated mice (Pearson *r* = − 0.85 with *P* = 0.0003). Future studies need to address the differential effects of MV settings and duration on BALF macrophage numbers and evaluate the exact role of macrophages in the development of VILI. Another negative correlation was found between lung wet to dry ratios and compliances in both MV groups. This finding supports the rationale that accumulation of interstitial and alveolar edema decreases compliance of the respiratory system as gas in small airways becomes displaced with fluid [37]. Lung compliance and wet to dry ratio were only altered in mice ventilated with HV_T_/ZEEP for 12 hours, which may reflect that more time is required for enhanced microvascular permeability and subsequent fluid filtration into the interstitial and alveolar space. 

The present study knows several limitations. First, clinically relevant V_T_ that closely reflect current MV practice in critically ill patients were used. Within this range of clinically relevant V_T_, we restricted the experimental design to a “less” and “more” injurious MV strategy (LV_T_/PEEP and HV_T_/ZEEP, resp.). Second, it has been described that mice have different respiratory mechanisms than humans [32, 33]. Moreover, smaller species have less resistance to VILI than larger species [34]. Therefore, a tidal volume of 7 mL/kg may have a greater effect in mice than in humans, where it is considered a protective ventilator setting. In addition, the lifespan of mice is much shorter compared to that of humans making 12 hours of MV relatively longer in mice than in humans. These differences in physiology may hamper the translation of current results to the human situation. Third, the analysis was restricted to some well-known characteristics of VILI such as the proinflammatory response, immune cell infiltration, alveolar-capillary permeability, and lung function. And fourth, the effects of MV were studied in otherwise healthy mice. The effects of longer duration of MV may be even more distinct in mice with lung injury.

## 5. Conclusions

In healthy mice, longer duration of MV aggravates important aspects of VILI compared to shorter-lasting MV or spontaneous breathing, with the phenotype of VILI changing over time. Furthermore, even less injurious ventilator settings may induce important aspects of VILI after 12 hours of MV. Thus, when interpreting data from animal studies, it is important to realize that deleterious effects of MV are dependent not only on its strategy but also on its duration.

## Figures and Tables

**Table 1:** Ventilator settings and arterial blood gas analysis.

	LV_T_		HV_T_	
	5 h	12 h	5 h	12 h
A				
*V*_T_	7.0 (6.8 to 7.2)	7.0 (6.8 to 7.6)	15.0 (14.9 to 15.4)	15.0 (14.8 to 15.2)
*P*_plat_	11.0 (10.3 to 12.0)	11.0 (11.0 to 12.5)	20.0 (18.5 to 21.0)	25.5 (22.0 to 26.8)*
B				
pH	7.48 (7.41 to 7.51)	7.34 (7.28 to 7.47)	7.43 (7.39 to 7.50)	7.46 (7.37 to 7.49)
PaCO_2_	32.3 (26.9 to 38.7)	42.4 (29.7 to 48.6)	32.9 (28.6 to 36.5)	31.2 (29.3 to 38.6)
BE	−1.6 (−2.3 to 2.7)	−2.9 (−4.7 to 1.7)	−1.9 (−4.5 to 2.4)	−2.1 (−3.3 to −0.4)
HCO_3_^−^	23.0 (19.0 to 25.6)	22.8 (20.3 to 23.7)	21.4 (19.0 to 25.7)	21.7 (20.4 to 22.7)

LV_T_, HV_T_ = ventilation with LV_T_/PEEP or HV_T_/ZEEP settings; 5 h, 12 h = 5 or 12 hours of ventilation; *V*_T_ = tidal volume in mL/kg; *P*_plat_ = plateau pressure in cmH_2_O; PaCO_2_ = partial pressure of arterial carbon dioxide in mmHg; BE = base excess in mmol/L; HCO_3_^−^ = bicarbonate in mmol/L. Data are presented as median (IQR) of 4–8 (A) or 11–15 (B) mice per group. *Illustrates primary statistical analysis (*P < 0.05 versus 5 hours).

**Table 2:** Total histopathology score.

	NVC	LV_T_		HV_T_	
		5 h	12 h	5 h	12 h
Score	1.0 (0.0 to 2.0)	1.5 (1.0 to 2.8)	1.0 (0.0 to 2.5)	3.0 (2.0 to 3.8)	4.0 (3.0 to 4.8)^∗∗, #^

NVC = nonventilated control; LV_T_, HV_T_ = ventilation with LV_T_/PEEP or HV_T_/ZEEP settings; 5 h, 12 h = 5 or 12 hours of ventilation. Data are presented as median (IQR) of 6–8 mice per group. *illustrates primary statistical analysis (**P < 0.01 versus NVC); ^#^illustrates secondary statistical analysis (^#^ *P* < 0.05 versus 12 h LV_T_).
